# A comparison of postoperative outcomes after open and laparoscopic reduction of Petersen's Hernia: a multicenter observational cohort study

**DOI:** 10.1186/s12893-021-01200-8

**Published:** 2021-04-15

**Authors:** Jae-Seok Min, Kyung Won Seo, Sang-Ho Jeong, Ki Hyun Kim, Ji-ho Park, Ki Young Yoon, Tae-Han Kim, Eun-Jung Jung, Young-tae Ju, Chi-Young Jeong, Ju-Yeon Kim, Young-Joon Lee

**Affiliations:** 1 Department of Surgery, Cancer Center, Dongnam Institute of Radiological and Medical Sciences, Busan, South Korea; 2 Department of Surgery, Kosin University Gospel Hospital, Busan, South Korea; 3 Department of Surgery, Gyeongsang National University School of Medicine, Jinju, South Korea; 4 Department of Surgery, Gyeongsang National University Hospital, Jinju, South Korea; 5 Department of Surgery, Gyeongsang National University College of Medicine and Gyeongsang National University Changwon Hospital, 11, Samjeongja-ro, Seongsan-gu, Changwon-si, Gyeongsangnam-do Republic of Korea

**Keywords:** Petersen’s hernia, Internal hernia, Laparoscopy, Gastric neoplasm

## Abstract

**Background:**

The aim of this multicenter cohort study was to compare the clinical courses between open and laparoscopic Petersen’s hernia (PH) reduction.

**Method:**

We retrospectively collected the clinical data of patients who underwent PH repair surgery after gastrectomy for gastric cancer from 2015–2018. Forty patients underwent PH reduction operations that were performed by six surgeons at four hospitals. Among the 40 patients, 15 underwent laparoscopic PH reduction (LPH), and 25 underwent open PH reduction (OPH), including 4 patients who underwent LPH but required conversion to OPH.

**Results:**

We compared the clinical factors between the LPH and OPH groups. In the clinical course, we found no differences in operation times or intraoperative bowel injury, morbidity, or mortality rates between the two groups (*p* > 0.05). However, the number of days on a soft fluid diet (OPH vs. LPH; 5.8 vs. 3.7 days, *p* = 0.03) and length of hospital stay (12.6 vs. 8.2 days, *p* = 0.04) were significantly less in the LPH group than the OPH group. Regarding postoperative complications, the OPH group had a case of pneumonia and sepsis with multi-organ failure, which resulted in mortality. In the LPH group, one patient experienced recurrence and required reoperation for PH.

**Conclusion:**

Laparoscopic PH reduction was associated with a faster postoperative recovery period than open PH reduction, with a similar incidence of complications. The laparoscopic approach should be considered an appropriate strategy for PH reduction in selected cases.

## Background

Internal hernia can occur as a result of the artificial mesenteric opening left by entero-entero anastomosis or Petersen’s space after a Roux-en-Y anastomosis. Petersen's hernia (PH) is rare but potentially fatal complication that can develop as the result of strangulation or perforation of a herniated small bowel [1]. Therefore, PH should be treated as soon as possible following its detection. Recently, it was reported that the incidence of internal hernia was higher after laparoscopic gastrectomy than after open gastrectomy, possibly because of poor adhesion after laparoscopic surgery [2].

Laparoscopic surgery is a recommended treatment modality for cancer (stomach, colon, liver, biliary) because it is associated with faster postoperative recovery, a lower incidence of complications, and better quality of life outcomes of patients than open surgery [3–8]. Laparoscopic internal hernia reduction in the small segment of the small bowel has been reported, but the surgical outcomes associated with laparoscopic PH reduction have not been reported [9, 10]. The aim of this multicenter cohort study was to compare the clinical courses between open and laparoscopic PH reductions. This is the first report involving a large multicenter cohort of gastric cancer patients after gastrectomy.

## Methods

A retrospective observational study was designed and carried out according to the principles of the Declaration of Helsinki, 1989. This study was approved by the institutional review board (approval number of first author’s institution, IRB-D-1909-009-002, and corresponding author’s institution, GNUH-IRB-2020-3-32).

### Patients

**Fig. 1:**
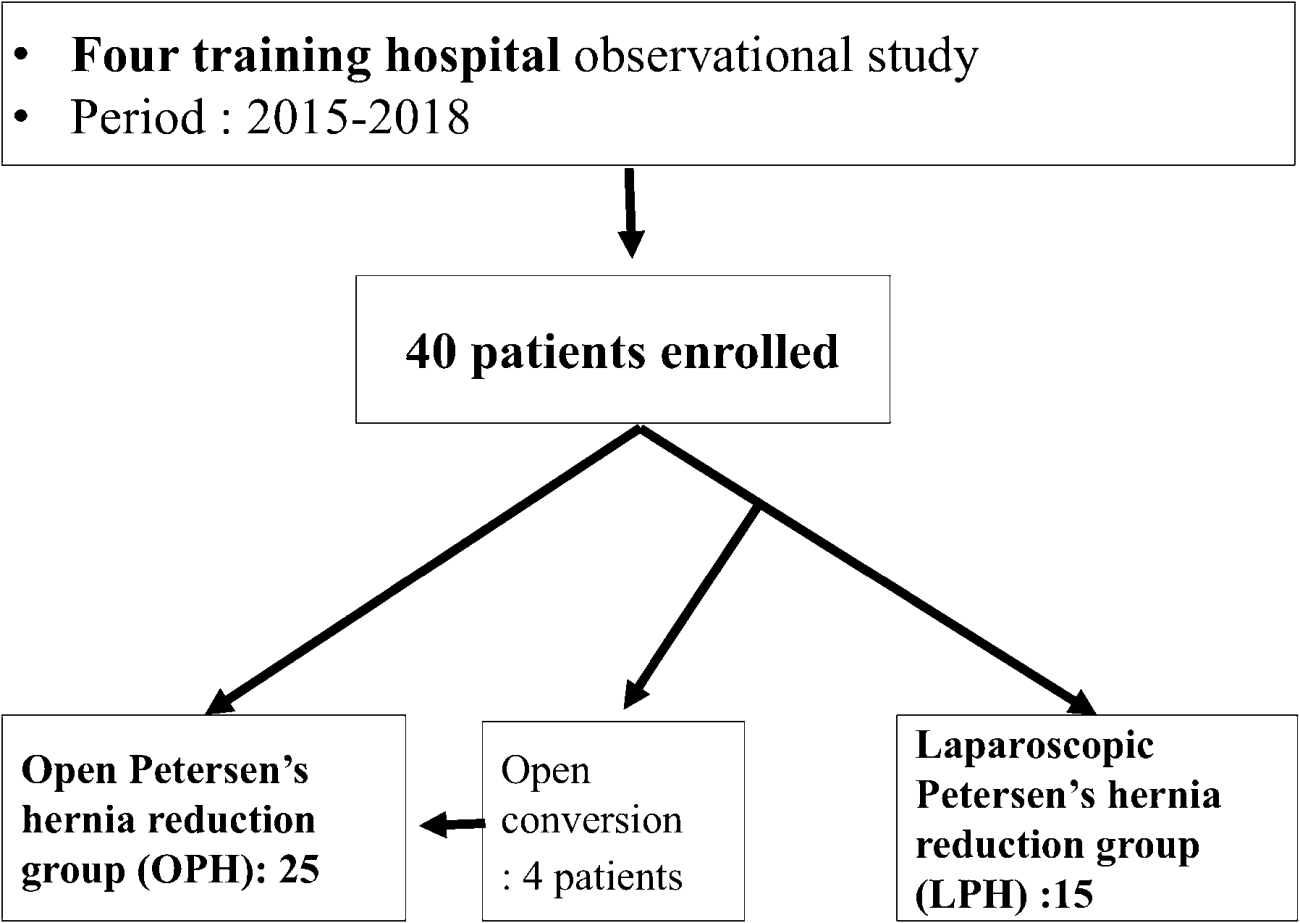
Diagram of patient enrollment

We retrospectively collected the clinical data of patients who underwent PH repair surgery after gastrectomy for gastric cancer from 2015–2018. Between 2015 and 2018, a total of 40 PH operations were performed at four hospitals (Changwon & Jinju Gyeongsang National University Hospitals, Dongnam Institute of Radiological and Medical Sciences, and Kosin University Gospel Hospital). Among the 40 patients, 15 underwent laparoscopic PH reduction (LPH), 4 underwent LPH with open conversion, and 21 underwent open PH reduction (OPH). Therefore, a total of 25 patients underwent OPH (Fig. 1).

Six expert surgeons have been meeting and sharing their experiences with LPH. All the surgeons were specialists in laparoscopic gastrectomy with experience in performing more than 200 cases.

The data of patients who underwent emergency surgery from 2015 to 2018 for mechanical ileus caused by PH were collected. The inclusion criteria for this study were as follows: (1) previous radical surgery for histologically proven gastric adenocarcinoma; and (2) no evidence of other distant metastases.

We collected data regarding the operation, postoperative complications, and course by retrospective chart review after surgery. We compared the past gastric cancer surgery method, TNM stage, surgical information, and postoperative course.

### Operations

#### Decision of open vs. laparoscopic reduction

The decision to perform laparoscopic reduction or open reduction was made by the surgeon after determining whether the patient had undergone laparoscopic surgery in the past, whether the patient presented with poor vital signs in the emergency room, and the statuses of bowel edema and dilation in the abdominal cavity on abdominal CT.

#### Open reduction

The operation began with an incision at the site of the previous incision, and an additional incision was made if necessary. During the incision, adhesiolysis was carefully performed to prevent injury to organs with adhesions. After the abdominal wall was opened, the viability of the herniated small bowel was determined. If the herniation direction was easy to detect, surgery was performed immediately. However, it was sometimes difficult to determine the correct direction of herniation. In these cases, we performed the following. First, we identified the ileocaecal valve and fully reduced the herniated bowel. After complete reduction, we checked the status of bowel perforation and necrosis. If the bowel was not viable even after reduction, we resected the damaged small bowel segment. We closed Petersen’s space with non-absorbable sutures.

#### Laparoscopic reduction

We inserted *t*he first trocar at just inferior to the umbilicus or in the lower abdomen area by an open method with a new incision. Mostly, we performed the reduction using three or four trocars. The intra-abdominal reduction process was the same as that performed in the open method. However, it was difficult to determine the correct direction of herniation with a laparoscope. Therefore, we identified the ileocaecal valve, traced the bowel proximally to identify the herniating segment then reduced it with gentle traction. After complete reduction, we checked the status of bowel perforation and necrosis. If the bowel was not viable even after reduction, we resected the damaged small bowel segment. We closed Petersen’s space with non-absorbable sutures (supplement Video).

The possibility of damage or bleeding in the small intestine is higher during laparoscopic hernia reduction than during open reduction due to forceful pulling of laparoscopic graspers. We recommend open conversion for patients with a poor pneumoperitoneum condition or severely damaged bowel, as reduction is difficult due to severe bowel edema.

### Statistical analysis

For statistical analysis, SPSS Statistics version 24 (IBM SPSS, Inc., Chicago, IL, USA) was used. Continuous data were compared using unpaired students' *t*-test and presented as ± standard deviations, and non-continuous variables were evaluated as chi square tests. For all analyses, *p* values below 0.05 were considered statistically significant.

## Results

### Patient demographics

Between 2015 and 2018, a total of 40 PH operations were performed at the four hospitals. The patient demographics are described in Table 1. The average age of patients was 63.9 years old, and the ratio of men to women was 4:1 (32:8). The TNM stage at past gastrectomy was as follows: the highest was stage I, which accounted for 60%; stage II accounted for 22.5%; stage III was the lowest and accounted for 5%; and unknown TNM stage accounted for 12.5%. Regarding past surgeries, there were 11 total gastrectomies (TGs), 24 distal gastrectomies (DGs) and 2 proximal gastrectomies (PGs). The past surgical methods comprised laparoscopic methods in 28 cases, open methods in 7 cases, and unknown methods in five cases. The mean period between operations was 28.2 months. The mean duration between the presentation of pain and hernia operation was 51.3 h.

**Table 1.** Patients demographics

Factors		Value
Age (years)		63.9 ± 12.1
Sex	Male	32 (80 %)
	Female	8 (20 %)
TNM stage of Gastric cancer*	I	24 (60%)
	II	9 (22.5%)
	III	2 ( 5%)
	Unknown	5 (12.5%)
Previous operation	TG with RNY-EJ	11 (27.5%)
	DG with B II	17 (42.5%)
	DG with RNY-GJ	2 (5%)
	DG with uncut RNY-GJ	5 (12.5%)
	PG with double tract	2 (5%)
	Unknown	3 (7.5%)
Previous op Approach method	Open gastrectomy	28 (70%)
	Laparoscopy assisted	7 (17.5%)
	Unknown	5 (12.5%)
Periods between previous operation (Month)		28.2 ± 45.7
Time duration between pain to hernia operation (hour)		51.3 ± 96.4
Approach method	Open reduction	21 (52.5%)
	Laparoscopic reduction	15 (37.5%)
	Open conversion of laparoscopic reduction	4 (10.0%)
Operation time		79.5 ± 25.1 minutes
Small bowel injury during reduction		4 (10%)
Hospital stay (day)		10.9 ± 6.8
Morbidity		4 (10%)
Mortality		1 (2.5%)

*AJCC TNM stage 8th edition, *GIST* gastrointestinal stromal tumor, *TG* total gastrectomy, *RNY* Roux-en Y, *EJ* esophagojejunostomy, *B II* Billroth II, *GJ* gastrojejunostomy, *PG* proximal gastrectomy

Regarding the approach for PH reduction, 15 patients underwent LPH, 4 patients underwent LPH with open conversion, and 21 underwent OPH. Therefore, a total of 25 were OPH (Fig. 1). The causes of open conversion were difficulty in creating a pneumoperitoneum (n = 2) and difficulty in laparoscopic reduction due to small bowel and mesentery thickness (n = 2). The mean operation time was 79.5 min; 4 patients experienced small bowel injury during reduction. The mean hospital stay was 10.9 days. The morbidity rate was 10% (4 cases), and there was one mortality.

### Comparison of clinical factors between the LPH group and the OPH group

We compared the clinical factors between the LPH and OPH groups. We found that the OPH group was older (68.8 years) than the LPH group (55.7 years) (*p* < 0.001) and had a higher C-reactive protein (CRP) level (OPH vs. LPH; 5.5 vs. 0.72, *p* = 0.03). However, there were no differences in sex, initial symptoms, duration between operations, TNM stage, previous operation, previous approach method, preoperative white blood cell (WBC) count, or erythrocyte sedimentation rate (ESR) (*p* > 0.05, Table 2).

**Table 2.** Clinicopathologic comparison between Laparoscopic reduction group (Laparo group) and open reduction group (Open group)

Factors		Open group (N=25)	Lapro group (N=15)	*P* value
Age (years)		68.8 ± 10.4	55.7 ± 10.4	<0.001
Sex	Male	19	13	0.68
	Female	6	2	
Initial symptom	Nausea	7	4	1.0
	Vomiting	8	5	1.0
	Pain	24	14	1.0
Periods between previous operation (month)		29.9 ± 56.5	25.4 ± 20.2	0.77
TNM stage of Gastric cancer*	I	14	10	0.13
	II	4	5	
	III	2	0	
	unknown	5	0	
Time duration between pain to hernia operation (hour)		40.0 ± 83.3	70.7 ± 116.4	0.35
Previous operation	TG with RNY EJ	7	4	0.50
	DG with B II	10	7	
	DG with RNY	1	1	
	DG with uncut RNY	2	2	
	PG with double tract	2	0	
	Unknown	3	0	
Previous approach method	Laparoscopy	15	13	0.20
	Open	6	1	
	unknown	4	1	
Preop Laboratory	WBC	10,025 ± 5.045	8,546 ± 2,768	0.30
	ESR	23.7 ± 25.2	16.3 ± 13.9	0.39
	CRP	5.5 ± 10.5	0.72 ± 1.1	0.03

*AJCC TNM stage 8th edition, *GIST* gastrointestinal stromal tumor, *TG* total gastrectomy, *RNY* Roux-en Y, *EJ* esophagojejunostomy, *B II* Billroth II, *PG* proximal gastrectomy, *WBC* white blood cell, *ESR* erythrocyte sedimentation rate, *CRP* C-reactive protein

### Comparison of operations and postoperative clinical courses between the OPH group and LPH group

We found no differences in operation time or intraoperative bowel injury, morbidity, or mortality rates (*p* > 0.05). However, the number of days on a soft fluid diet (OPH vs. LPH; 5.8 vs. 3.7 days, *p* = 0.03) and length of hospital stay (12.6 vs. 8.2 days, *p* = 0.04) were significantly less in the LPH group than in the OPH group (Table 3).

**Table 3.** Comparisons of operation data and postoperative clinical courses between the open reduction group (Open group) and laparoscopic reduction group (Laparo group).

Clinical factor	Open group (N=25, %)	Lapro group (N=15, %)	*P* value
Operation time	83.1 ± 26.1	73.6 ± 23.1	0.25
Bowel injury during operation	2 (8%)	2 (13.3%)	0.62
SFD start day	5.8 ± 3.1	3.7 ± 2.1	0.03
Hospital stay	12.6 ± 7.4	8.2 ± 4.9	0.04
Morbidity	2 (8%)	1 (6.7%)	1.0
Mortality	1 (4%)	0	1.0

*ASIS* anterior superior iliac spine, *SMV* superior mesenteric vein

### Postoperative complications

In OPH group, one case of pneumonia and one case of sepsis with multi-organ failure resulted in mortality. In the LPH group, one patients experienced recurrence of PH at postoperative day 8, and he underwent a second reduction by the open method (Table 4). The patient was discharged 16 days after reoperation without other complications.

**Table 4:** . Postoperative complications

Complications	Open group	Lapro group
Pneumonia	1	0
Sepsis with multi-organ failure	1	0
Petersen’s hernia recur	0	1

## Discussion

The aim of this multicenter cohort study was to compare the clinical courses between open and laparoscopic **PH** reductions**. Laparoscopic reduction is not always possible, but if so then** the postoperative recovery course is generally better, and operative wounds are generally smaller with the laparoscopic approach than with open surgery. However, to the best of our knowledge, **no one has reported a comparison between laparoscopic and open PH reduction**. The present multicenter observational cohort study is the first to report **some** advantage of laparoscopic PH reduction in terms of postoperative recovery.

PH is a very rare disease that occurred in only 0.54% of patients who underwent gastrectomy for gastric cancer (2417; the number of patients requiring PH reduction surgery was 13) between 2015 and 2018. In a previous study, the incidence of PH after gastrectomy was 0.42%, which was similarly low compared to our data [2, 3]. For PH reduction surgery, the open method is the standard procedure due to the following: first, it is difficult to develop pneumoperitoneum due to bowel edema and bowel dilation in the abdominal cavity; second, bowel reduction is difficult due to bowel wall and mesentery edema, and usually the whole small bowel is herniated; third, experience in laparoscopic PH reduction is limited [2, 9, 11]. In our group, the first laparoscopic reduction was performed in 2014 in Kosin University Hospital; since then, gastric experts from the four centers share their experiences with laparoscopic PH reduction during regular meetings. Sharing their experiences facilitates the application of laparoscopic PH reduction because all surgeons are gastric cancer surgery specialists with experience in performing more than 200 laparoscopic gastrectomies.

Laparoscopic surgery has advantages. We found that the number of days on a soft fluid diet and postoperative hospital length of stay were significantly less in the laparoscopic reduction group than in the open group. The reason for the faster recovery is likely that laparoscopic surgery resulted in fewer operative wounds and less pain than open surgery. Additionally, if the bowels were not manipulated excessively by the surgeon during laparoscopy, a postoperative diet could be permitted quickly, causing a potential decrease in the length of hospital stay.

Recently, the application of the laparoscopic approach for primary gastrectomy has increased; however, one study reported the possibility of an increased incidence of internal hernia [2]. They reported that laparoscopic TG was associated with a higher incidence of internal hernia than open TG (4.5%, 29/638 vs. 0.8%, 4/475), with the same results for laparoscopic DG compared with open DG (2.7% vs. 0.9%). In the multivariate analysis, they found that non-closure of mesenteric defects, the laparoscopic approach, and a total laparoscopic approach were independent risk factors for internal hernia. We expected the incidence of PH to be higher in primary laparoscopic gastrectomy patients; however, the incidence of PH was higher in primary open gastrectomy patients. This may be because more patients underwent open gastrectomy than laparoscopic gastrectomy in the past. Even though there was only a small number of PH cases included in this study, interestingly, the laparoscopic or open primary approach for gastrectomy did not affect the incidence of PH. In contrast, a recent study reported that closure of the mesenteric space (5.5%) significantly decreased the cumulative incidence of reoperation for small bowel obstruction by internal hernia after surgery compared to no mesenteric closure (10.2%) [12]. Therefore, to reduce postoperative complications, especially the incidence of internal hernia after gastrectomy surgery, the closure of mesenteric defects should be performed [2]. The centers included in this study routinely repair mesenteric defects after gastrectomy and have recently started performing closure of Petersen’s space to prevent PH.

In terms of postoperative complications, pneumonia generally occurs more frequently in patients who undergo open surgery; open surgery patients also experience more wound pain than patients who undergo minimally invasive surgery [7]. In this study, the one pneumonia patient was an 80-year-old individual who was at increased risk for postoperative complications due to age. In the laparoscopic group, a complication of internal hernia recurrence after reduction surgery was observed. This suggests that we need to close defects more carefully because reduction and closure after laparoscopic surgery is more difficult than after manual open surgery.

There were some limitations to our study, such as the small number of PH reduction surgeries especially laparoscopic reduction surgeries. All the surgeons participating in the present study were specialists in gastric cancer surgery with experience in performing more than 200 laparoscopic gastrectomies. However, the surgeons did not have many chances to face the cases of Petersen’s hernia which needed a reduction surgery, because the incidence of Petersen’s hernia is very low. In addition, since this is a retrospective observational cohort study, there might be a selection bias in determining the surgical approach method before surgery. The patients were not randomized in choosing their surgical approach. The surgeon might have chosen patients with easy recovery as a laparoscopic approach method. Nevertheless, to the best of our knowledge, this is the first report comparing the surgical outcomes associated with open and laparoscopic approaches for PH reduction.

## Conclusions

Laparoscopic PH reduction was associated with faster postoperative recovery and a similar incidence of complications compared with open surgery. The laparoscopic approach should be considered an appropriate strategy for PH reduction in selected cases.

## Data Availability

Not Applicable.

## Abbreviations

PH: Petersen’s hernia
LPH: Laparoscopic Petersen’s hernia reduction
OPH: Open Petersen’s hernia reduction
TG: Total gastrectomy
DG: Distal gastrectomy
PG: Proximal gastrectomy
CRP: C-reactive protein
WBC: White blood cell
ESR: Erythrocyte sedimentation rate
